# Sentiment Analysis toward the COVID-19 Vaccine in the Main Latin American Media on Twitter: The Cases of Argentina, Chile, Colombia, Mexico, and Peru

**DOI:** 10.3390/vaccines11101592

**Published:** 2023-10-14

**Authors:** Alba Córdoba-Cabús, Manuel García-Borrego, Yaiza Ceballos

**Affiliations:** Department of Journalism, Faculty of Communication Sciences, University of Malaga, 29071 Málaga, Spain; manoletus@uma.es (M.G.-B.); yaiza.ceballos@uma.es (Y.C.)

**Keywords:** vaccine, vaccination, COVID-19, coronavirus, sentiment analysis, Latin America, media, Twitter, journalism, digital media

## Abstract

This article analyzes the media coverage of the COVID-19 vaccine by major media outlets in five Latin American countries: Argentina, Colombia, Chile, Mexico, and Peru. For this purpose, the XLM-roBERTa model was applied and the sentiments of all tweets published between January 2020 and June 2023 (*n* = 24,243) by the five outlets with the greatest online reach in each country were analyzed. The results show that the sentiment in the overall media and in each nation studied was mostly negative, and only at the beginning of the pandemic was there some positivity. In recent months, negative sentiment has increased twelvefold over positive sentiment, and has also garnered many more interactions than positive sentiment. The differences by platform and country are minimal, but there are markedly negative media, some more inclined to neutrality, and only one where positive sentiment predominates. This paper questions the role of journalism in Latin America during a health crisis as serious as that of the coronavirus, in which, instead of the expected neutrality, or even a certain message of hope, the media seem to have been dragged along by the negativity promoted by certain discourses far removed from scientific evidence.

## 1. Introduction

The coronavirus pandemic, caused by severe acute respiratory syndrome coronavirus-2 (SARS-CoV-2), emerged in December 2019 in China, with its epicenter being the City of Wuhan. Following the assessment of the disease’s magnitude and its disturbing levels of spread, the World Health Organization (WHO) declared coronavirus disease 2019 (COVID-19) a pandemic on 11 March 2020. The first Latin American country to identify a positive case was Brazil, on February 25 in São Paulo, an area that, given its expanse and connections, had already experienced significant outbreaks of infections, such as with virus Zika in 2016 [1,2].

Latin America as a whole has been severely affected by COVID-19, especially by the weakness and precariousness of its health infrastructure, high prevalence of chronic diseases, poverty, and inequalities, as well as by the problems stemming from its particular political situation [3,4]. Faced with this health crisis scenario, the various responses of the different countries in the region have been aligned according to their social, cultural, and economic contexts [5].

In Mexico, the first case of coronavirus was identified on 27 February 2020, and measures such as the suppression of non-essential activities, social distancing, and lockdown were implemented to try to slow its advance. In Chile, the first positive test was reported on 3 March 2020, and the government established, among other policies, mandatory quarantines and curfews, as well as a unification of the public and private health systems. On the same day, the first case of COVID-19 was reported in Argentina, where measures such as the closure of cultural spaces and borders and the issue of licenses for remote work were decreed. Peru registered its first positive test on 6 March 2020, and 10 days later the government imposed quarantine and curfew restrictions. The coronavirus also arrived in Colombia on March 6, and the measures implemented were along the same lines. In other words, in this aspect, the region acted similarly and presented unity.

However, despite this unity against COVID-19′s arrival, vaccination in the region has been exceptionally varied. While in Chile, Mexico, and Argentina the vaccination campaign began in December 2020, in Peru and Colombia it did not start until February 2021. This has resulted in widely disparate vaccination rates, with Chile as the country with the highest vaccinated population rate (90.3%), Mexico the lowest (63.1%), and Argentina (83.1%), Peru (82.4%), and Colombia (70.5%) in intermediate positions [6]. Dreser [7] argues that low vaccination rates are due to the fragility of health systems—for example, lack of personnel and resources such as deep freezers for storage—and corruption in governments—for example, in Mexico, Peru, and Chile, theft of vaccines and vaccination of non-priority groups were discovered. Misinformation also undermined the success of vaccination campaigns, with hoaxes about the genetic damage that vaccination could cause or the supposed injection of microchips during inoculation [8].

In crisis situations such as this, information becomes an essential resource, serving not only to know what is happening around us, but also as a mechanism to guide the population [9]. In this sense, the media play a decisive role as intermediaries between institutions and citizens [10,11,12], as they can contribute toward reducing uncertainty and facilitating adaptation to the new reality [13]. Moreover, during these periods of instability, the need to be informed, and consequently, the social relevance of the media increases [14,15,16,17,18,19,20,21,22].

In this ecosystem, social networks play a key role, as the media opt for the dissemination of content through these types of platforms with the intention of expanding their sphere of influence [23,24,25,26]. Social networks have completely changed the information consumption habits of citizens: they have become the preferred channel of information for 30% of the world’s population [27], with Facebook being the most used application for this purpose (28%). Furthermore, several authors consider Twitter to be the most efficient social network in the field of communication [28,29,30], estimating that it is the application that concentrates the largest number of people interested in current affairs [31,32] and is the one that journalists use most frequently [33]. The potential of Twitter lies in its universality, immediacy, ease of interaction, and participation among users, as well as the possibility of disseminating content without the need to rework it [34,35,36].

### 1.1. Analysis of Sentiments toward COVID-19 and the Vaccine

There are numerous authors who analyze Twitter and propose sentiment analysis on this platform as a technique for monitoring attitudes, thoughts, and opinions regarding a particular product or event [37,38,39,40,41,42], and in particular for understanding the perception of the population in crisis situations [43]. Thus, this tool has already been used by several authors during the coronavirus crisis to ascertain the sentiment of the general population [44,45,46,47,48,49,50], with disparate results depending on the context, the object of study, and the model used, and both positive [48] and negative [49,51] reactions depending on the study’s characteristics.

In relation to the COVID-19 vaccine, Yousefinaghani et al. [52] analyzed the sentimental progression of tweets over a year (between January 2020 and January 2021) and found a predominance of positive sentiment in the messages of the population, although how the anti-vaccine movement begins to gain strength with the declaration of the pandemic in March 2020, with relevant peaks in April and December, can also be seen. In Jalil et al. [53] also, the positive tone prevails, with most of the messages being aimed at commenting on the safety and effectiveness of vaccines, which is in line with Agrawal et al. [44]. In addition, according to Hussain et al. [54], positive messaging toward the vaccine generated a higher volume of interactions. 

Ruiz-Núñez et al. [55], in a literature review, discovered similar findings. These authors analyzed messages distributed on Twitter by both pro-vaccine and anti-vaccine bots and conclude that pro-vaccine content generates higher levels of interaction. Furthermore, it has been observed that on Twitter, research results, vaccination data, and practical information are more closely associated with a positive attitude toward vaccines, while news-related content tends to elicit a neutral response [56].

In line with the aforementioned, other authors, such as Christensen et al. [57], have noted that during the pre-pandemic period, media coverage of vaccines, in general, was relatively marginal and characterized by significant negativity (57% of messages). However, with the onset of the pandemic, the topic of vaccines shifted to a prominent position on the media agenda and received more positive treatment (with only 38% of messages being negative). Nevertheless, the perception of negativity increased due to the greater volume of content published. One of the challenges associated with vaccine-related negativity is that, at times, the media turns to pseudoscientific sources that disseminate false information to the public [58]. Finally, there are other authors who have analyzed the sentiment generated on Twitter regarding the vaccine in 192 countries and arrive at different conclusions, as they assert that messages with a negative tone prevail. [59]. Moreover, Jemielniak and Krempovych [60] add that the most frequently retweeted tweets often contain negative information and, in many cases, originate from media sources known for disseminating misinformation.

Vaccine sentiment among the general population has also been studied in several Latin American countries [61,62,63,64]. In Colombia, Rodríguez-Orejuela et al. [65] emphasized the abundance of negative content, mainly expressed as fear and anger. The most bitter months were July, August, and September 2021, coinciding with the reinfection of people already vaccinated and with the limitations of a vaccine in the experimental phase. In Ecuador, Xavier et al. [66] reported wide acceptance of the vaccine during the first national immunization campaign. Salcedo-Lagos et al. [64] focused on as many as nine Spanish-speaking countries—Argentina, Chile, Ecuador, El Salvador, Spain, Mexico, Panama, Peru, and Venezuela—and identified a particularly negative vaccine burden, with widespread distrust of scientific and governmental authorities in eight of the nine countries examined. In order of highest to lowest negativity, Argentina is in the first position, followed by Mexico, Panama, Spain, Venezuela, El Salvador, Ecuador, Peru, and finally Chile.

### 1.2. Coverage of the Coronavirus Crisis

Academia has also addressed the media treatment of this health crisis [9,22,64,65], since its impact has greatly transformed the way journalism is produced and consumed, and media have become an essential element in the process of citizen and democratic decision-making. Generally speaking, traditional media, especially television [9] and digital media [22], have been found to be more prominent than social networks.

Several studies have been carried out in Latin America. In Argentina, Zunino and Arcangeletti-Yacante [67] analyzed the media coverage of COVID-19 during the first 150 days of the pandemic and found that the coronavirus monopolized the media agenda: it was present in six out of ten published news items. The public debate ranged from purely sanitary issues, promoted by official information, to political discussion on the consequences of lockdown. Likewise, Zunino [68] observed a clear predominance of official sources, although they became partisan coverage, during the period of isolation in Argentina.

In other countries, such as Mexico, the perception of risk in the face of the virus increased the level of dependence on the media system [69,70]. During the first phase of the pandemic, the population did not perceive coronavirus as a threat, a feeling that changed as the pandemic progressed. According to the authors, this change was mainly due to the effects of exposure to news on television and digital media as well as interpersonal conversations. In Ecuador, López et al. [71] analyzed the credibility of the information content on COVID-19 and evidenced the loss of trust in the traditional media when they promoted themselves utilizing partisan interests. As a result, they ascertained a clear preference for information from digital media, international television, or social networks when the content was shared by family and friends. Along the same lines, Greene-González et al. [72] dissected media messages in Colombia and determined that they have a positive tone when they are about entertainment and a negative tone when they are informative or come from social networks, mainly due to the presence of information saturation and the distrust generated by fake news.

### 1.3. Objectives

As evident, the media treatment and the polarity of the messages issued on the coronavirus crisis in general and on the vaccine in particular constitute an issue of enormous importance, since to a large extent they can condition the understanding and acceptance of society. Thus, the sentiment analysis technique as a tool for analyzing the direction of messages regarding the COVID-19 pandemic has been widely employed by academia [44,45,46,47,48,49,50]; however, these investigations have focused only on messages delivered by the public and not by the media itself. Given the need for a general understanding of the attitude of the media toward vaccines and the feelings provoked by their messages, this article has two fundamental objectives:

Objective 1 (O1): To describe the media coverage related to the COVID-19 vaccine in Latin America through the media with the greatest online reach in Argentina, Chile, Colombia, Mexico, and Peru;

Objective 2 (O2): To examine differences in media coverage according to dates, country, platform, and media outlet.

## 2. Methodology

In order to study the emotional charge of the main Latin American media reports on Twitter toward the COVID-19 vaccine, five Spanish-speaking countries were chosen on the basis of population criteria [73] and the availability of information consumption records: Argentina, Chile, Colombia, Mexico, and Peru [27]. Specifically, the five media outlets that have the greatest online reach in quantitative terms, according to the Digital News Report [27], were selected for each nation:

- Argentina: A24 online (non-governmental), *Clarín online* (non-governmental), *Infobae* (non-governmental), *La Nación online* (non-governmental), and TN online (non-governmental).
- Chile: 24horas online (governmental), Chvnoticias.cl (non-governmental), *Emol.com* (non-governmental), *Laterecera.com* (non-governmental), and Meganoticias.cl (non-governmental).
- Colombia: *El Espectador online* (non-governmental), *El Tiempo online* (non-governmental), *Las 2 Orillas* (non-governmental), Noticias Caracol TV online (non-governmental), and *Pulzo* (non-governmental).
- Mexico: *Aristegui News* (non-governmental), *El Universal news online* (non-governmental), Televisa news online (non-governmental), TV Azteca news online (non-governmental), and UnoTV news online (non-governmental).
- Peru: *El Comercio online* (non-governmental), *La República online* (non-governmental), Latina Noticias (non-governmental), *Perú21 online* (non-governmental), and *RPP News online* (non-governmental).

The sample consisted of all messages related to the coronavirus vaccine that were published between 31 December 2019 at 00:00 and 1 June 2023 at 23:59 on the official Twitter accounts of the selected media (*n* = 24,243). We decided to assess the sentiment towards the vaccine since the arrival of the COVID-19 to understand the news context prior to its inoculation.

### 2.1. Collection and Processing of Information

R statistical software (version 4.2.2) was used for data collection. The tweets were downloaded on June 14 through the Twitter Academic API (v2)—limit 10 million tweets/month–, using the academictwitteR library. The variables collected and generated were the following: user, date of publication, public metrics such as number of retweets, likes, quotes and replies, and raw text (Table 1). As a query, only the original tweets from the selected media were requested, thus avoiding retweets from other accounts, if there were any, and which included the association of the word “vaccine” with “COVID” and/or “coronavirus”. Subsequently, links, videos, images, and mentions were removed from each tweet using the Python programming language.

### 2.2. Analysis

After data cleaning, a sentiment analysis was carried out to identify the emotional charge of each of the texts. For this purpose, the “twitter-XLM-roBERTa-base for Sentiment Analysis” model, a natural language processing algorithm trained on 198 million tweets published between May 2018 and March 2020 in more than 30 languages, was applied [74]. XLM-roBERTa combines the strengths of XLM, focused on learning linguistic representations through machine translation, which allows for the detection of common grammatical patterns and structures in different language, and RoBERTa, an optimized variant of the BERT model, able to understand the context of words in a sentence. It is a multilingual algorithm, with sentiment adjustment in eight languages widely used in text classification, tagging, summary generation, and machine translation tasks, among others.

The performance of the model varies depending on the language, but the results for Spanish are very robust in massive data analysis (around 85% accuracy), showing a higher rate than that of others such as VADER (76.8%) or TextBlob (68.8%). XLM-roBERTa calculates the probability that a message is positive, negative, or neutral. In this research, messages with a probability of more than 50% of being positive were considered positive, identical to the mechanism applied for the negative case. Tweets that had more than a 50% chance of being neutral, or did not reach 50% on any of the sentiments, were categorized as neutral.

## 3. Results

### 3.1. COVID-19 Vaccines in the Latin American Media: A General Overview

**Figure 1:**
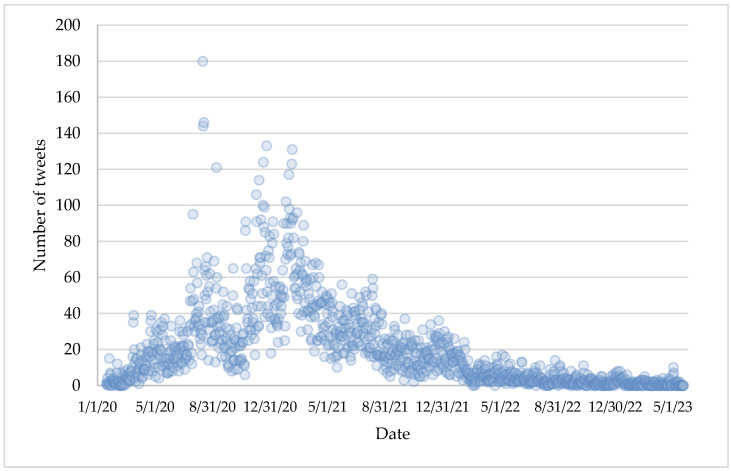
Number of tweets about COVID-19 vaccine per day (2020–2023).

A total of 24,243 tweets were downloaded from the selected media. The first of these date from the week of 20–26 January 2020, with China reporting that it was already working on the COVID-19 vaccine, and the last from 18 May 2023, referring to information from the WHO on the protection offered today by the vaccines administered. The bulk of messages are concentrated in the first two years of the pandemic, 2020 (40.3% of the sample) and 2021 (51.1%), while in 2022 and 2023, the figures dropped considerably (7.8% and 0.9%, respectively). Figure 1 shows the distribution of tweets on each of the days during the 3.5 years studied. The peak was around 11 August 2020, when Putin announced the Sputnik vaccine, the first to enter the development phase.

The print media were much more active on Twitter than the television stations: they accounted for 73.0% of the messages downloaded, but more importantly, they published an average of 1263 tweets compared with 637 from the television stations. The newspaper with the highest number of items was *El Comercio*, from Peru, with 3245 items. Peru was also by far the country with the highest number of tweets published by the reference media: 8830 (36.4% of the sample) compared with 4237 (17.5%) from Argentina, which is in second place.

With regard to the analysis of sentiment, it was observed that 14.8% of the messages published by the selected media conveyed negative ideas about the vaccine, while positive sentiment were the majority in only 6.6%. In other words, more than twice as many negative tweets were posted about the vaccine than positive ones, while 78.6% remained neutral.

**Figure 2:**
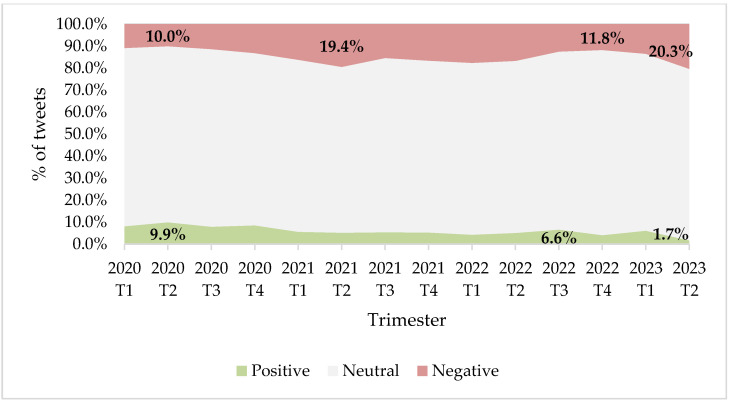
Sentiment of tweets about COVID-19 vaccine by trimester (2020–2023).

Figure 2 shows the quarterly evolution of each of the sentiments between January 2020 and June 2023. The highest point of positive messages is in the second trimester of 2020, with almost the same proportion of tweets favorable toward the vaccine (9.9%) as unfavorable ones (10.0%). This is a phase of relative hope, with early clinical trials reportedly beginning to show good results in adults, but even at this point, the most positive, pro-vaccine sentiment does not outweigh the negative.

This first phase of good reception of the vaccine leads to the bad second trimester of 2021, when the campaign to administer the doses begins and the presence of the anti-vaccine movement intensifies, which starts to talk about the alleged side effects such as magnetism. At this stage, negative sentiment toward the vaccine permeates almost one-fifth of all tweets posted (19.4%). Subsequently, the negative sentiment moderates slightly, but then gains momentum to reach its strongest point precisely in the last trimester studied, between 1 April and 31 May 2023, when various controversies linked to the vaccine, such as the price increases defended by pharmaceutical companies or the death of one of the creators of Sputnik, come to light. Currently, negative tweets about the vaccine are up to 12 times more common than positive ones.

Positive information about the vaccine has a similar social media impact to negative information: the average number of retweets is slightly higher (18.6 to 17.2), and the average number of likes is much higher (78.6 to 51.1); however, replies and quotes, which are usually associated with disagreement on the part of users, are lower (7.2 to 11.7 in replies and 3.2 to 4.9 in quotes). Neutral publications, meanwhile, have fewer retweets (10.6), likes (42.8), and quotes (2.7) on average, while replies have similar numbers (7.7).

**Figure 3:**
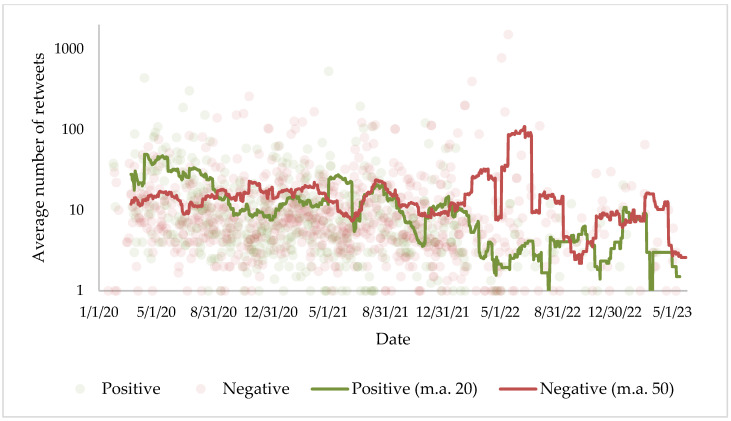
Average number of retweets per day according to the sentiment of the tweet and moving average of the last 50 days.

More striking is the evolution of interactions (see Figure 3): in the first 9 months of the pandemic, the number of retweets of positive information about the vaccine exceeded those of negative information, but at the end of 2020, the trend began to change, and since 2020, negative messages about the vaccine have systematically exceeded positive ones in terms of interactions.

### 3.2. Vaccine Sentiment by Country, Medium, and Platform

Table 2 presents the results stratified by country and platform. In the first block, it can be seen how Peru, the country in which the media have dedicated the most tweets to the COVID-19 vaccine, is also where the positive sentiment is lower (5.9%), with figures similar to Colombia (6.5% positive), the nation with the most negative tweets (15.7%). Mexico, meanwhile, is the country with the most positive (7.7%), least negative (13.3%), and most neutral (79.1%) tweets, although negative coverage also predominates. As for the type of platform, the differences seem hardly noticeable, since both positive (6.6%) and negative (15.4%) information is emphasized in the press, with less than two percentage points of distance in each of the sentiments. No radio data are provided, as there is only one radio station in the sample (RPP Noticias in Peru).

**Figure 4:**
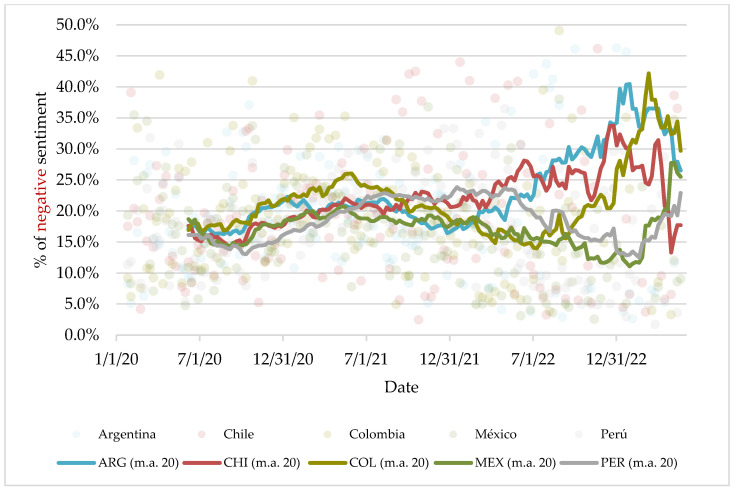
Percentage of negative sentiment by week, country, and moving average from the last 20 weeks of analysis.

The historical series (Figure 4) shows how, at the beginning of the pandemic, the greatest negative sentiment toward the vaccine was consistently prevalent in Colombia, but from 2022 onward, Chile, and especially Argentina, lead in unfavorable coverage toward the vaccine, while Mexico almost always remains slightly below the other countries. In all countries, the greatest fluctuations have been observed in recent months, which is in line with the reduction in the volume of information on the vaccine; as it is no longer part of daily coverage, it mainly appears when more extreme events occur, in all senses of the word.

**Figure 5:**
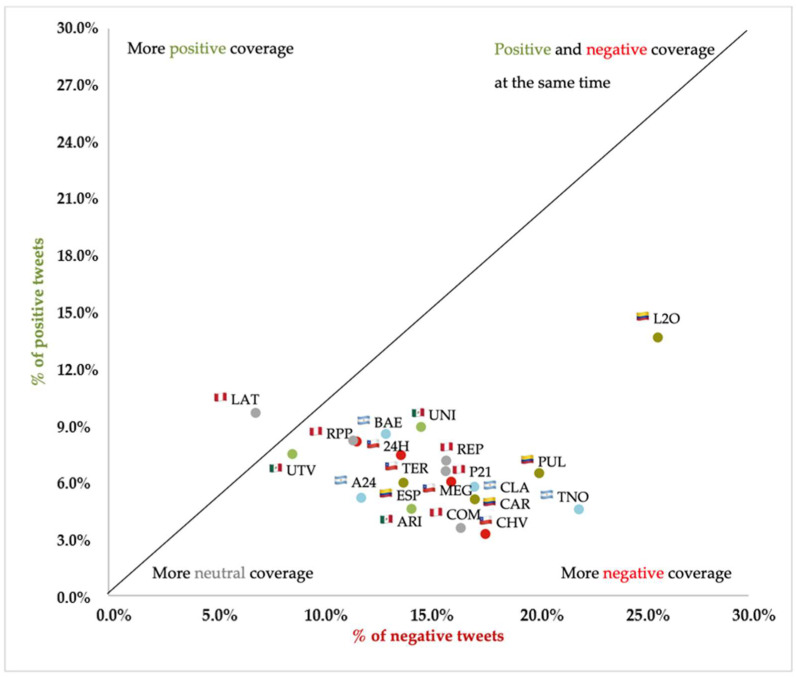
Percentage of positive and negative tweets by medium.

The biggest difference seems to be between the media themselves. Figure 5 shows the position of each media outlet according to the percentage of positive and negative tweets. The media outlet with the most unfavorable coverage of the vaccine is Todo Noticias (TNO), from Argentina, with 22.1% negative tweets and only 4.6% positive ones (that is, almost five times less). CHV Noticias in Chile, *Pulzo* (PUL) in Colombia, *Aristegui Noticias* (ARI) in Mexico, and *El Comercio* (COM) in Peru are the media with the most negative coverage in their respective countries.

At the other extreme is Latina Noticias (LAT) in Peru, with 9.7% positive tweets and 6.9% negative ones. It is the only media outlet that has published more positive information about the vaccine than negative, as evidenced by the line crossing the figure. A24 in Argentina, 24 Horas (24H) in Chile, *El Tiempo* (TIE) in Colombia, and Uno TV (UTV) in Mexico are close behind. The medium with the most extreme values is *Las 2 Orillas* (L2O) from Colombia, which is the one with the most positive tweets dedicated to the vaccine (13.6% of all those published), and at the same time, the one with the most negative tweets (25.8%). *Emol.com* (Chile), Televisa (Mexico), and TV Azteca (Mexico) were omitted from the analysis as none of them had more than five tweets in 3.5 years.

## 4. Discussion and Conclusions

The results obtained in this research present strong connections with previous studies, given the high number focused on the coronavirus vaccine and citizens’ perceptions of its administration [44,52,53,61]. However, above all, the results allow us to delve into a hitherto barely explored aspect: the media coverage of the main Latin American media using sentiment analysis.

The first objective of this article was to describe, in broad outline, the media coverage of the COVID-19 vaccine using the reference newspapers of the five Latin American countries included in the Digital News Report [27]: Argentina, Chile, Colombia, Mexico, and Peru. As expected, it was seen that the vaccine that could be the remedy to the coronavirus pandemic became a top item on the news agenda, reaching an average of almost ten tweets per account per day at its peak, in line with the findings of Zunino and Arcangeletti-Yacante [67]. Likewise, it was also observed that the written press has been the main source of publications on the vaccine, with an average of more than 1200 for each entity studied.

However, coverage has been far from positive, as shown with the sentiment analysis carried out. Among the nearly 25,000 tweets analyzed, negative sentiment was more than twice as high as positive sentiment, which raises certain doubts as to the role of the media in the fight against disinformation caused by the anti-vaccine movement, and is conflated with the negativity expressed by the users themselves [63,64], perhaps in a logical response to the information they consume. Although it is true that the figures obtained should be taken with relative caution—the model utilized also records information not directly related to the effects of the vaccine as negative, such as, for example, the death of one of Sputnik’s developers in a domestic dispute, as reported by the media—this does not imply that there is not a certain dissonance between what is expected of information on a vaccine called to combat and remedy a global pandemic and what was finally found: negativity, and in the best of cases, neutrality. Nonetheless, we want to emphasize this consideration as a potential limitation inherent to the sentiment analysis model we applied. It primarily distinguishes between positive, negative, and neutral sentiments without delving into the various nuanced emotions within each category. Nevertheless, given that the data are sourced from media outlets, this categorization scheme still allows us to conduct a meaningful analysis of COVID-19 vaccine media coverage.

The evolution of sentiments and interactions over time reinforces the thesis that the anti-vaccine discourse has strengthened over the course of the pandemic: positive sentiment toward the vaccine has plummeted in the media from 2020 to the present date—today it is six times less likely to find a tweet favorable to any of the vaccines than during lockdown—and the audience of these media seems to have changed its own preferences; while at the beginning positive tweets were disseminated and shared to a greater extent, since 2022 the dynamics have changed radically and publications associating the vaccine with negative sentiments have gained prominence in the face of disinterest in positive information. These results are consistent with the contributions of other studies [46,49,50,51,75]: as the pandemic has progressed, the fear of the disease and the projection of anti-vaccine discourse have grown, and with it the negativity surrounding the immunization process.

Although this phenomenon seems to be generalized, it is difficult to establish patterns by country or platform, as suggested by O2, and the differential factor is to be found, above all, in the media themselves.

By country, Mexico has maintained a somewhat more favorable attitude toward vaccination than countries such as Colombia and Peru, but the differences are minimal, always below two percentage points in each of the sentiments, which suggests that, in practice, the differences are not so pronounced and that this depends largely on the nature and proportion of the media included in the sample. Something similar happens with the platforms: the differences between the press and television are hardly worth mentioning.

The responsibility for the results would therefore lies on the editorial line followed by each headline. There are firms such as Todo Noticias in Argentina characterized by publishing almost a quarter of messages with negative sentiments, a proportion five times higher than the positive coverage of the vaccine. This contrasts with the work of A24 or *Infobae* in Argentina, whose sentimental load is more evenly distributed. Despite this, negative sentiment is also in the majority in these two cases.

Strikingly, only one of the media studied published more positive than negative news about the vaccine: Latina Noticias in Peru. However, there are also examples in Peru such as *El Comercio*, which publishes five negative tweets for every positive one, balancing the country’s results. Similar realities are repeated in Chile, Colombia, and Mexico, although the media are less polarized in their positions. The results of *Las 2 Orillas*, the media outlet that shares the most positive, and at the same time, the most negative news about the vaccine—perhaps as a result of trying to make their content go viral—and those of *Emol.com*, Televisa, and TV Azteca, which despite their position in the Digital News Report [27], have only dedicated three, zero, and five tweets, respectively, to the COVID-19 vaccine, deserves a special mention.

The pandemic has not only caused a health crisis of enormous magnitude, but has also secondarily fed the virus of disinformation, which journalism must try to combat by employing the utmost rigor and facilitating society’s decision making through information that is, in this case, based on scientific evidence. The results presented in this study raise serious doubts as to the work of the main newspapers in Latin America, which, instead of reporting in an eminently neutral manner and highlighting positive information when progress is made —which in the context of a pandemic and in the case of vaccines, should be the predominant one— and unfavorable information when there are complications, seem to have been carried away by the negative current. Only in the long term will it be possible to determine the real effects of this type of information coverage.

## Figures and Tables

**Table 1:** List of variables.

Variables	Type
User	String
Date of publication	DateTime
Number of retweets	Scale
Number of likes	Scale
Number of quotes	Scale
Number of replies	Scale
Raw text	String

**Table 2:** Number of tweets by country and platform and percentage of positive, negative, and neutral sentiment.

Country	*N*	Positive	Neutral	Negative
Argentina	4237	7.2%	78.0%	14.9%
Chile	3265	6.6%	79.0%	14.5%
Colombia	3778	6.5%	77.8%	15.7%
Mexico	4133	7.7%	79.1%	13.3%
Peru	8830	5.9%	78.9%	15.2%
**Platform**	***N***	**Positive**	**Neutral**	**Negative**
Press	17,688	6.6%	78.1%	15.4%
Television	5099	6.3%	80.0%	13.7%

## Data Availability

The data that support the findings of this study are available here: https://gitlab.com/uma2/COVID19_vaccine_latinamerican_media/ (accessed on 13 October 2023).
